# A novel biocompatible Eu-based coordination polymers of cytarabine anticancer drug: Preparation, luminescence properties and *in vitro* anticancer activity studies

**DOI:** 10.3389/fchem.2022.1043810

**Published:** 2022-11-11

**Authors:** Zhijun Zeng, Huaxiang Shen, Wei Gao, Qifeng Guo, Mengjie Chen, Xiaojun Yan, Hongning Liu, Yanhua Ji

**Affiliations:** Research Center for Differention and Development of TCM Basic Theory, Jiangxi Province Key Laboratory of TCM Etiopathogenisis, Jiangxi University of Chinese Medicine, Nanchang, China

**Keywords:** Eu-based coordination polymers (Eu-CP-Ara), cytarabine, luminescence properties, anticancer drug, cytotoxicity

## Abstract

In this study, we use cytarabine anticancer drug to synthesize a new rare earth complex with Europium ion. The study work is an attempt to investigate luminescence and biological properties of the Eu-based coordination polymers of cytarabine (Eu-CP-Ara) anticancer drug which have been prepared by us. Eu-CP-Ara has luminescence properties with emission centering at about 619 nm excited with 394 nm. We study cytarabine and Eu-CP-Ara *in vitro* cytotoxicity. Cytotoxicity of Eu-CP-Ara against lung cancer cells (A549) could even be comparable to the inhibitory effect of cytarabine ligands, showing the advantage of antitumor activity. In addition, Eu-CP-Ara showed lower cytotoxicity to normal liver cells (L02). At the same, from the CLSM images, Eu-CP-Ara has successfully entered the A549 cell. Hence, Eu-CP-Ara can be used as a potential anticancer drug. Eu-CP-Ara may be an effective strategy for the tracking cytarabine against tumours and might impart better accurate treatment effect and therapeutic efficiency.

## Graphical Abstract

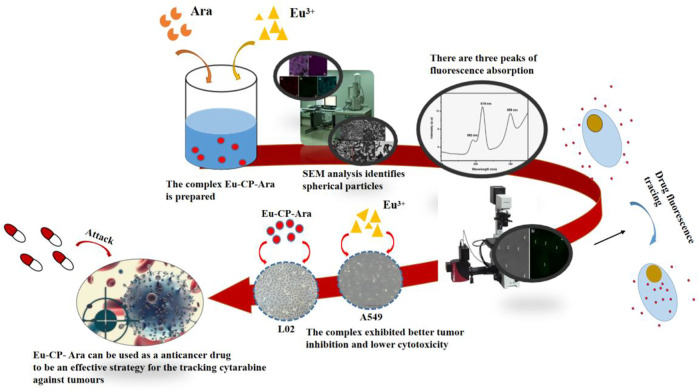

## Introduction

Cancer has disturbed human livelihood and health for a long time, which is one of the most serious diseases (Shu et al., 2021; Wuttke et al., 2015; Zhang and Zhao, 2018). Cytarabine (Ara-C) is an anticancer drug used for chemotherapeutic agent, which can treat the acute myeloblastic leukemia, individualized by abnormal proliferation of myeloid blasts in hematopoietic stem (Hirsch et al., 2017; Liu et al., 2018). Sometimes, cytarabine is also used as combination treatment drug to treat disease with other therapeutic method. For example, Low-dose cytarabine could combine exchange transfusion (ET) to treat Down syndrome without developing acute leukemia, liver failure, or other serious adverse events (Okamura et al., 2019). High-dose cytosine arabinoside combine autologous hematopoietic stem cell transplantation to treat mantle cell lymphoma (Cakar et al., 2020; Nato et al., 2021; Pola et al., 2021). Cytarabine (cytosine arabinoside 1-β-D-Arabinofuranosylcytosine, Ara-C) is a pyrimidine nucleoside analog, which could combine other anticancer drugs to treat solid tumors and show superbly synergistic effects against (Jabbour et al., 2007). For example, the combination of decitabine, idarubicin, and cytarabine chemotherapy drugs can treat acute myeloid leukemia and high-risk myelodysplastic syndrome (Zhang and Guo, 2019). This therapeutic method can improve survival of elderly patients and reduce early mortality (Kantarjian et al., 2012; Zhang and Guo, 2019). Cytarabine unite with sequential dosing of decitabine used in the treatment of acute myeloid leukaemia in children (Kearns et al., 2019). This novel therapeutic strategy is more effective than either agent alone in relapsed and refractory (Leonard et al., 2014; Kearns et al., 2019). Therefore, cytarabine anticancer drug are very important and useful in treating acute myeloid leukaemia. However, it has many severely drawbacks such as rapid plasma metabolism indicating a short half-life and low lipophilicity (Liu et al., 2018), which are extremely limiting clinical applications. Cytarabine has a certain inhibitory effect on acute leukemia and other cancer cells, but the anti-tumor effect of single cytarabine is limited, and its anti-tumor activity and various biological activities can be enhanced by forming complexes with metals.

In 1965, the American scientist Rosenberg’s group study revealed that cisplatin had anti-tumor activity and it showed a wide range of anti-cancer biological activity. This research results has led many scientists to pay attention to Inorganic-Organic Hybrid metal complexes, especially for the anticancer drug properties and activity of metal complexes (Alinaghi et al., 2020; Holmes, 2015; Pettinari et al., 2020; Shen et al., 2020; Zhang and Zhao, 2018), such as new imidazolium-based palladium (II) saldach complexes as potential anticancer agents to treat cancer patients (Alfaifi et al., 2019). Cisplatin which has anti-cancer activity also showed nephrotoxicity and neurotoxicity in biomedicine (Ameri et al., 2022). Discovering low-toxicity and high-efficiency anti-cancer drugs is the focus of many researchers. The coordination polymers have a rapid development in these years. At the same time, lanthanide coordination polymers have gained great attentions in recent years. Lanthanides, yttrium, and scandium are collectively referred to as rare earth elements. It has been reported that rare earths can regulate the aquaporins of plants and prevent the damage of cell membranes by active oxygen in the body, which can increase the water content of cells and achieve the effect of promoting plant growth and drought resistance (Salgado et al., 2020; Vorob’ev et al., 2019). Rare earths can affect the metabolism of hormones in the body, regulate the endocrine system, and increase the activity of enzymes (He et al., 2003; Schwabe et al., 2012). Because lanthanide coordination polymers not only have unique applications, but also own potential applications in biomedicine (Wang et al., 2018; Garcia-Valdivia et al., 2020; Liu et al., 2020), such as antitumor, antimicrobial, anticoagulant action, antivirus, which have been researched in recent decades (Liu and Yang, 2009; Trusova et al., 2013; Kant and Maji, 2021). In addition, lanthanide coordination polymers have good luminescence properties. Nowadays, many coordination polymers drugs, nanoparticles and materials have been used for anticancer activity study (Abanades Lazaro et al., 2018; Shen et al., 2018; Usman et al., 2021). For example, Eu(III)-based coordination polymer nanoparticles were investigated to anti-oral cancer studies (Zhang and Zhao, 2018). There are many reports on Eu(III)-based coordination polymer used in physical aspects such as structure and fluorescence (Arrué et al., 2021; Horniichuk et al., 2021; Khanagwal et al., 2021). However, Eu(III)-based coordination polymer used to anticancer drug are still fewer to report.

In terms of coordination polymer synthesis, there are heating reflux (Eftekhari far and Nasr-Esfahani, 2019), hydrothermal method (He et al., 2017; Fan et al., 2018), microwave radiation method (Ibarra-Vázquez et al., 2019), ultrasonic method (Razmara and Poorsargol, 2019), and solvothermal method developed from hydrothermal synthesis (Long et al., 2019), *etc.* In this work, Eu-based coordination polymers of cytarabine anticancer drug have been prepared under solvothermal conditions. We used cytarabine as the ligand that is an anticancer drug, europium (III) as the ligand metal, which possess interesting luminescent properties. These luminescent properties are conducive our to observe anticancer drug situation. We synthesized the coordination polymers that the morphologies are sphere-like with the size about 100 nm-2.5 μm. To date, few papers have been reporting this synthetic method. Therefore, the sphere-like product will be discussed herein.

## Materials and methods

### Materials

Eu(NO_3_)_3_·6H_2_O (purity: 99.99%) were purchased from the city of Shanghai Aladdin Industrial Corporation (China). Cytarabine were also purchased from the city of Shanghai Macklin biochemical technology (China). All aqueous solutions were prepared using Milli-Q 10 water (resistivity>18 MΩ cm). All chemical reagents and solvents used in this work were of analytical grade and were used without further purification.

### Instruments

FEI Quant 250FEG scanning electron microscopy (FEI Company, United States). ARL 3000 Desktop XRD Analyzer (Jingong Instrument (Suzhou) Co., LTD, French). ESXTAR6000 TG/DTA Thermal Difference Analyzer (Japan Seiko Electronics Nano Technology Co., LTD, Japan). Nicolet IS5 infrared spectrometer (Nicolet, United States). LEICA TCS STEDCW Ultra high-resolution microscope system (Leica Microsystems, Germany). HITACHI F-7000 Fluorescence spectrophotometer (Hitachi Scientific Instruments (Beijing) Co. LTD, Japan).

### Preparation of Eu-based coordination polymers of cytarabine anticancer drug

In this work, Eu-CP of cytarabine anticancer drug micro/nanospheres were prepared for the first time. In a typical synthesis procedure, 0.3 mmol of cytarabine anticancer drug was placed in a 20 ml Teflon-lined stainless-steel autoclave. Then it was dissolved in 10 ml absolute methanol under magnetic stirring. Then, 0.3 mmol Eu^3+^ was added to the above solution. Finally, we used triethylamine to adjust the PH of the above solution until the PH value was about 7. The autoclave was sealed and heated at 160°C for 6 h. The final products were collected by centrifugation and washed several times with ethanol and distilled water. The precipitate was dried at 60°C for 10 h in the end.

### Cytotoxic activities experiment *in vitro*

The tested compounds were dissolved in sterile water and diluted to the required concentration with culture. After the required concentration of solution was configured, 0.22 μm microporous membrane filter was used for filtration and sterilization. A549 cells were grown in F12 medium supplemented with 10% freshly inactivated fetal calf serum and antibiotics. L02 cells were grown in RPMI-1640 medium supplemented with 10% freshly inactivated fetal calf serum and antibiotics. The cells harvested from exponential phase (1 × 10 ^5^per ml) per well in 100 μl of medium were seeded equivalently into a 96-well plate and then incubated for 6 h at 37°C and 5% CO_2_, then the tested compounds were added in a concentration gradient, and the final concentrations were, respectively, maintained at 96, 48, 24, 12, 3, 1.5, 0.5 μg/ml. The plates were kept at 37°C in a humidified atmosphere of 5% CO_2_ and incubated for 48 h, the diluent of MTT of an appropriate concentration was added to each well and the plates incubated at 37°C and 5% CO_2_ for 4 h. Then suck the supernatant were added 110 μl formazan solution to each well, put on a shaking table and oscillated at low speed for 10 min to fully dissolve the crystals. The measurements of absorbance of the solutions related to the number of live cells were performed on an ELISA spectrophotometer at 450 nm.

### Data processing and statistical analyses

The results of the experimental data are expressed as mean ± standard error of mean. The differences between the two groups were analysed by *t*-test; the differences between multiple groups were compared by one-way analysis of variance using the software program R (http://www.r-project.org/). *p* < 0.05 was considered statistically significant.

## Results and discussion

### Characterization of micro/nanoparticles

**FIGURE 1:**
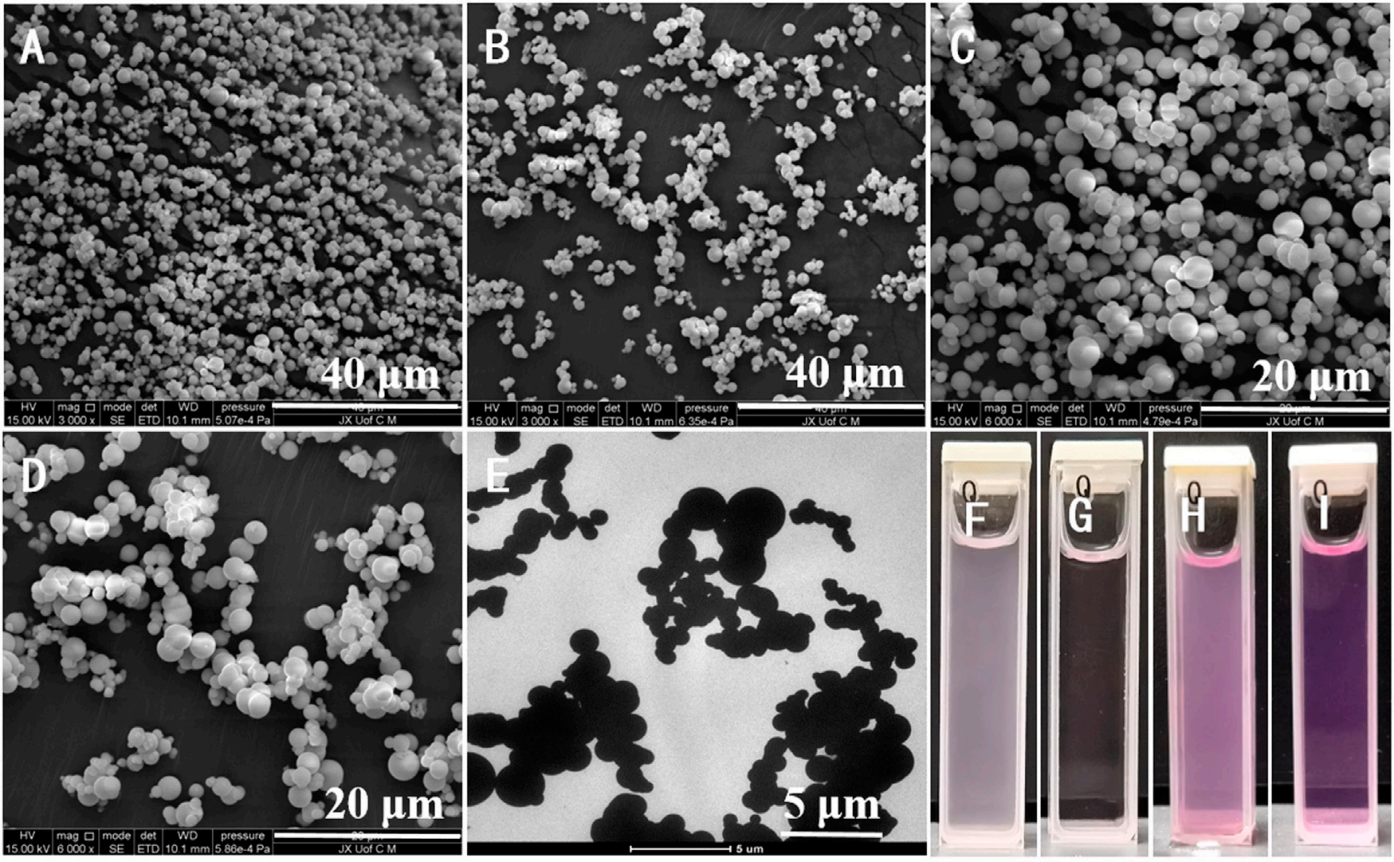
Surface morphology evaluation of the developed Eu-CP-Ara by SEM at different magnifications **(A–D)**. TEM images of Eu-CP-Ara micro/nanosphere assembly. The scale micro/nanosphere of the inset is 100 nm∼2.5 μm **(E)**. Eu-CP-Ara micro/nanosphere with a concentration of 200 μg ml^−1^ after 6 days **(F,H)** and the above solution obtained after centrifuging at 10,000 rpm **(G,I)**.

The SEM and TEM image with sphere-shape characterization shows that complex 1 prepared by solvothermal method has the average size around 100 nm∼2.5 μm (Figures 1A–E). The SEM image of developed Eu-CP-Ara revealed uniform, smooth surface, and spherical shape (Figure 1). The amplifying TEM reveal that Eu-CP-Ara is solid spherical shape. Moreover, this product showed good dispersibility when Eu-CP-Ara was dispersed in F12 medium (Figure 1F) gand RPMI-1640 medium (Figures 1H,I). After 6 days, the dispersed solution was still very stable (Figures 1F–I).

**FIGURE 2:**
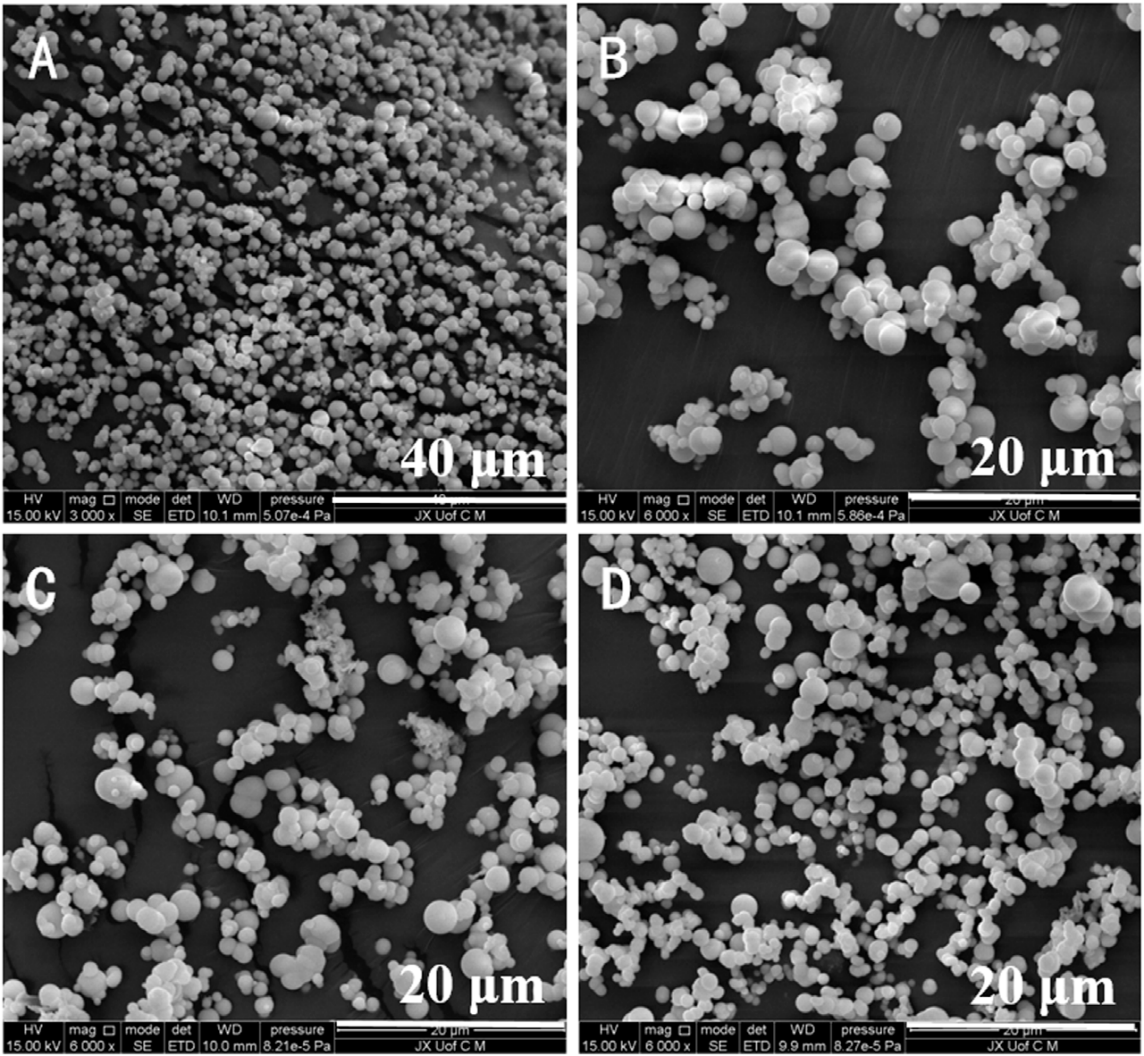
SEM images of Eu-CP-Ara micro/nanosphere after response time for 24 h **(A,B)**, 12 h **(C)** and 6 h **(D)**.

It has been reported that different reaction time has great influence on the morphology of products (Ji et al., 2020). So the stability of the Eu-CP-Ara micro/nanosphere was tested in different reaction time and recorded by SEM observation. The SEM image show that Eu-CP-Ara micro/nanosphere was very stable (Figure 2). The morphology of products did not change shape and remained spherical.

**FIGURE 3:**
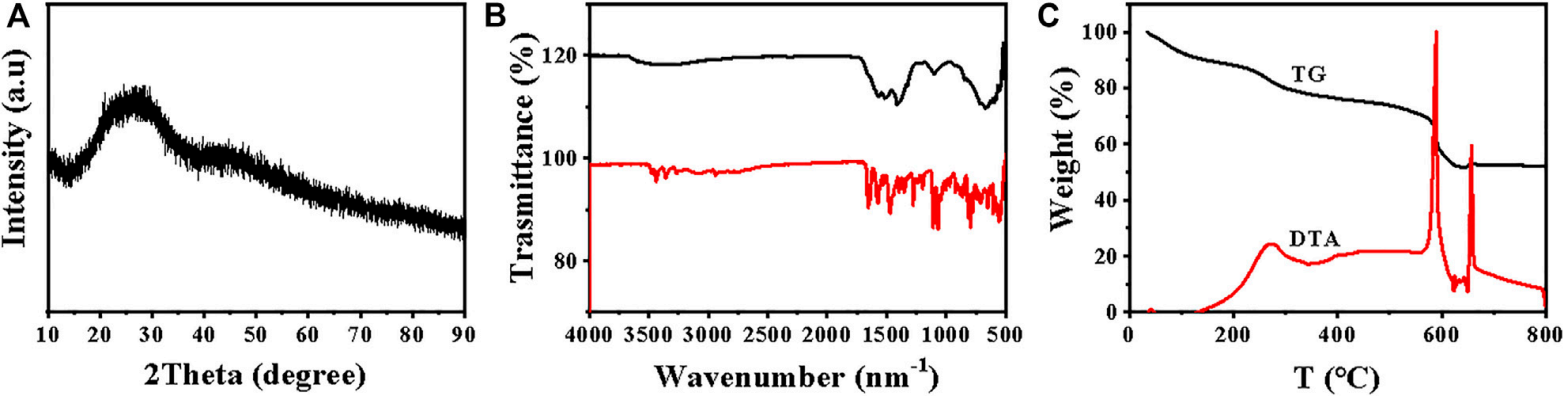
**(A)** XRD pattern, **(B)** IR spectrum and **(C)** TG-DTA curves of Eu-CP-Ara (Complex 1).

XRD pattern of the Eu-CP-Ara is shown in Figure 3A. It obviously displays that the Eu-CP-Ara is amorphous. The peak at 3,476 cm^−1^ and 3438 cm^−1^ are due to the antisymmetric stretching vibration and symmetric stretching vibration of amino group (Figure 3B). The peak at 3,355 cm^−1^ is show the absorption peak of oxyhydrogen stretching vibration (Figure 3B). By comparing the infrared image of cytarabine with its complexes, the amino peak and hydroxyl peak of cytarabine at 3,476 cm^−1^, 3,438 cm^−1^, and 3,355 cm^−1^ have disappeared. But it is shown in Figure 3B that a wide peak at 3,425 cm^−1^ is the stretching vibration of coordinate bond exists. The FT-IR results also confirms the formation of coordination polymer. TG and DTA curves of Eu-CP-Ara (Figure 3C) were also detected under atmospheric conditions at 800°C.TG analysis displays that the first mass loss in the range of 50–200°C is 12.0%, manifesting the loss of the physically absorbed water molecules. Two apparent decompositions between 200°C to 570°C and 575°C–665°C of the weight loss are ascribed to the cytarabine and Eu-CP-Ara frameworks.

**FIGURE 4:**
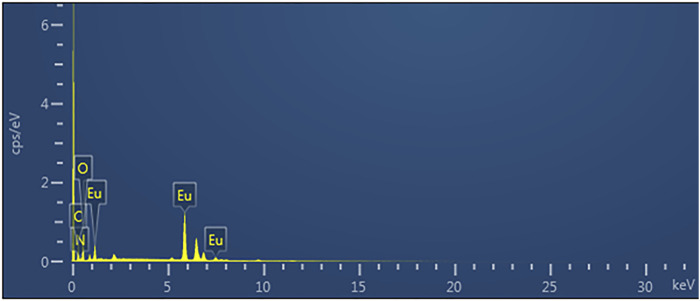
EDS diagram of Eu-CP-Ara.

**FIGURE 5:**
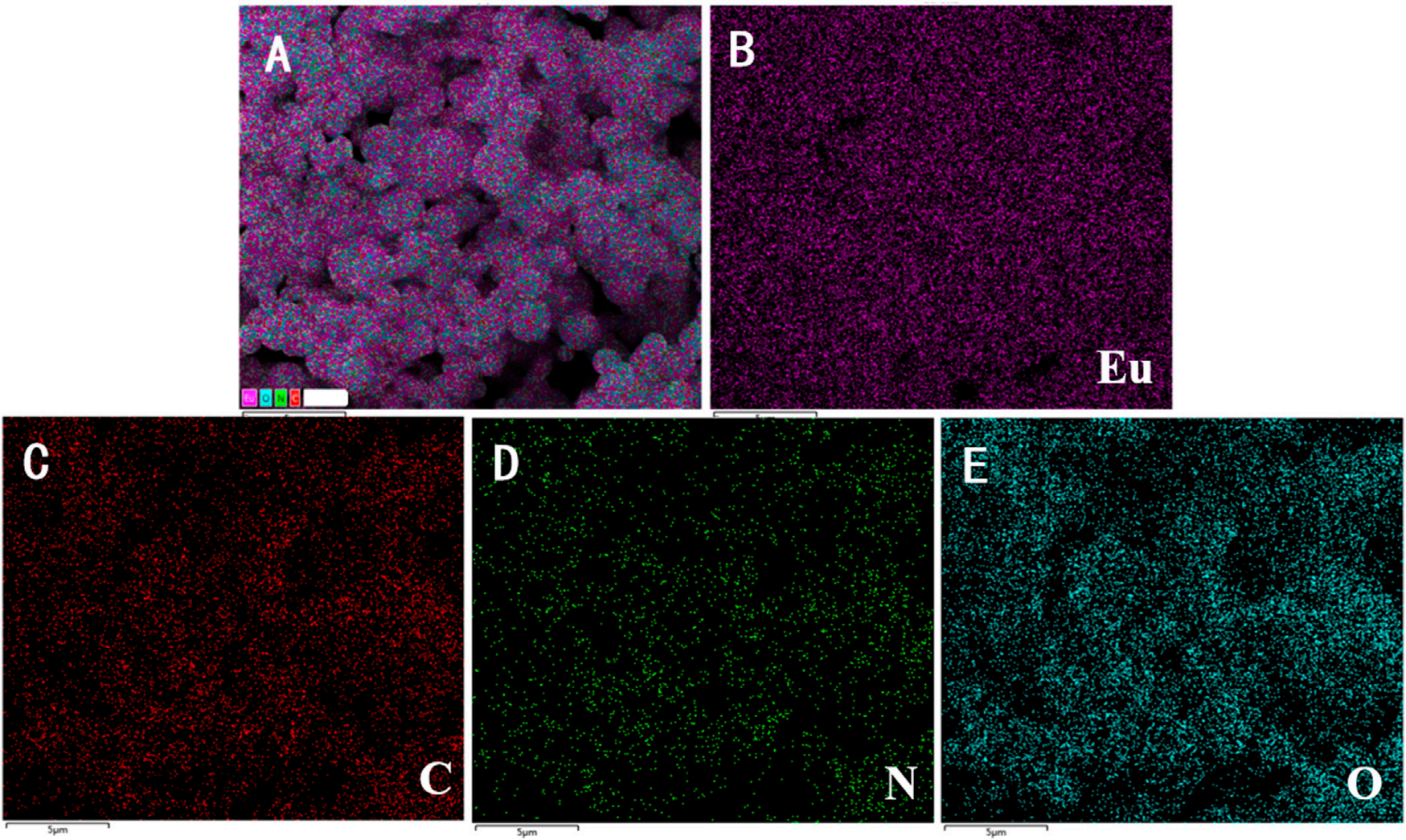
Elemental mapping images (EDS) of Eu-CP-Ara for **(A)** all the elements; **(B)** Eu; **(C)** C; **(D)** N; **(E)** O.

Figure 4 shows elemental mapping of Eu-CP-Ara micro/nanoparticle, it can be seen that the sphere-shape micro/nanoparticle consists of Eu, C, N, and O. The distribution of nitrogen is less (Figure 5D). In addition, the uniform distributions of Eu (Figure 5B), C (Figure 5C), N (Figure 5D), and O (Figure 5E) in Eu-CP-Ara sphere-shaped were further evidenced by EDS images in Figure 5. Elementary analysis shows that the percentage of europium content is 25.90%, the carbon content is 27.53%, the nitrogen content is 10.71%, and the oxygen content is 35.86%. Based on above results, the product can be proposed to be 2Eu·3(C_9_H_13_N_3_O_5_)·8(H_2_O).

### Photoluminescence spectroscopy of micro/nanoparticles

**FIGURE 6:**
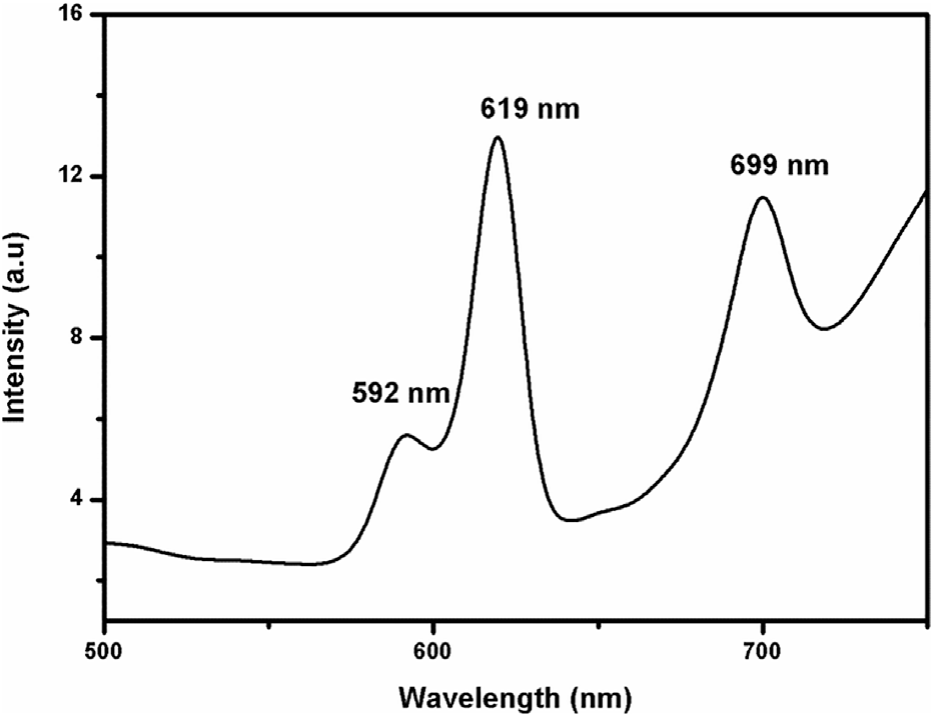
Emission spectra of Eu-CP-Ara particles under 394 nm excitation.

The emission spectra of the Eu-CP-Ara excited with 394 nm were shown in Figure 6, From the emission spectrum of Eu-CP-Ara particle, these Eu-CP-Ara particles show the three peaks located in 592 nm, 619 nm, 699 nm were all the typical emission of Eu^3+^ ions, the Eu^3+^ ions exhibit dominant red emission ascribed to ^5^D_0_→^7^F_1_, ^5^D_0_→^7^F_2_, ^5^D_0_→^7^F_3_ transitions of Eu^3+^.

### Cytotoxic activity of the micro/nanoparticles

**FIGURE 7:**
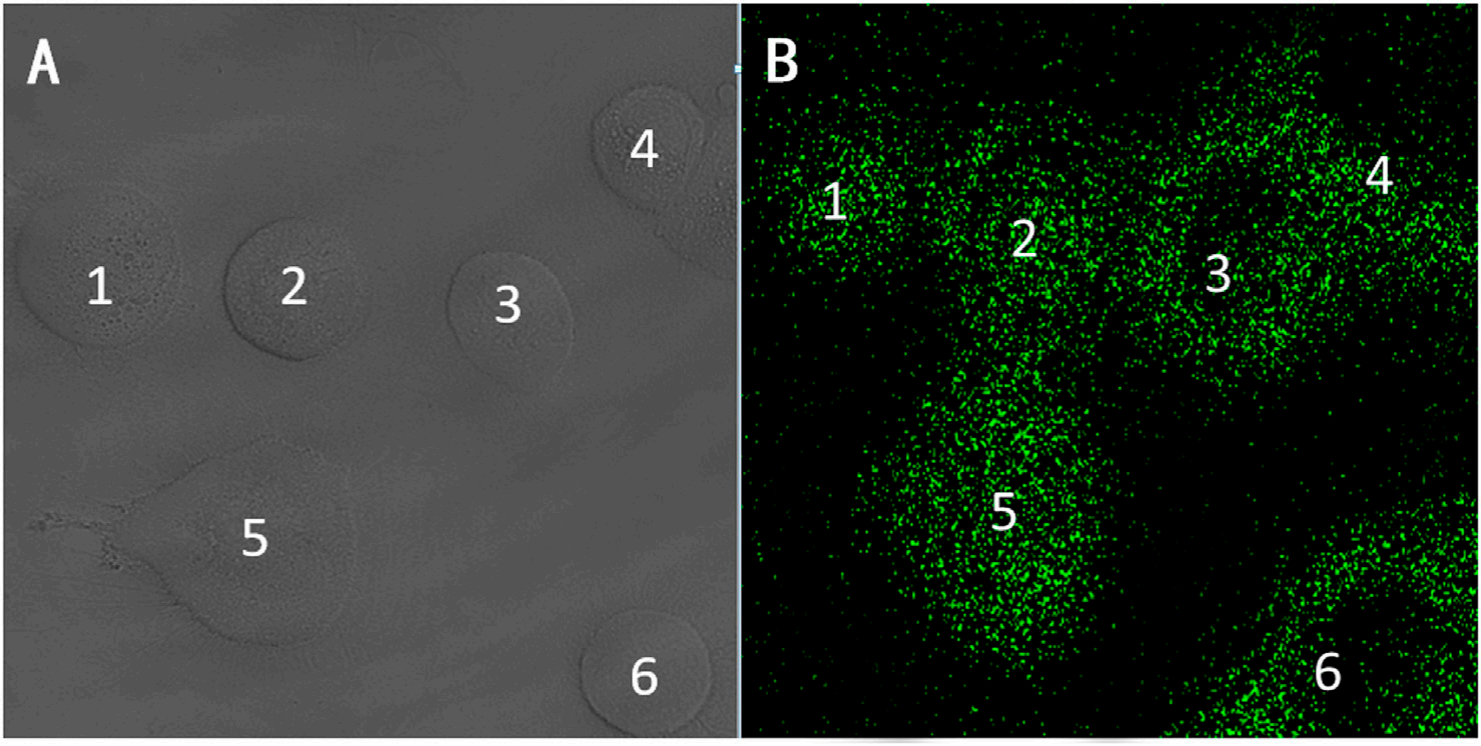
CLSM images after incubating A549 cells with Eu-CP-Ara. **(A)** Distribution of Eu-CP-Ara in A549 cells at the bright field image for 24 h. **(B)** Distribution of Eu-CP-Ara cells at the dark field image for 6 h after incubating A549 cells with Eu-CP-Ara.

As shown in Figure 7, the A549 cells uptake situation were detected by CLSM (Zou et al., 2017; Cheng et al., 2019; Lu et al., 2020). To understand the result of cell death in this Eu-CP-Ara, we investigated the intracellular distribution of the Eu-CP-Ara under a confocal laser scanning microscopy (CLSM) (Figure 7). From the CLSM images, the fluorescence intensity in green color could be clearly seen in the cell. Eu-CP-Ara has successfully entered the A549 cell and was distributed throughout the cell. In order to prove this conclusion, the CLSM images was utilized to further confirm the distribution of Eu-CP-Ara at the light field image (Figure 7A) and the results are shown in Figure 7B. The fluorescence of Eu-CP-Ara was mostly overlapped with that of the corresponding A549 cells. The results suggested that Eu-CP-Ara tended to enter in A549 cells and had good biocompatibility (Ji et al., 2021; Zeng et al., 2019; Zhang et al., 2022).

**FIGURE 8:**
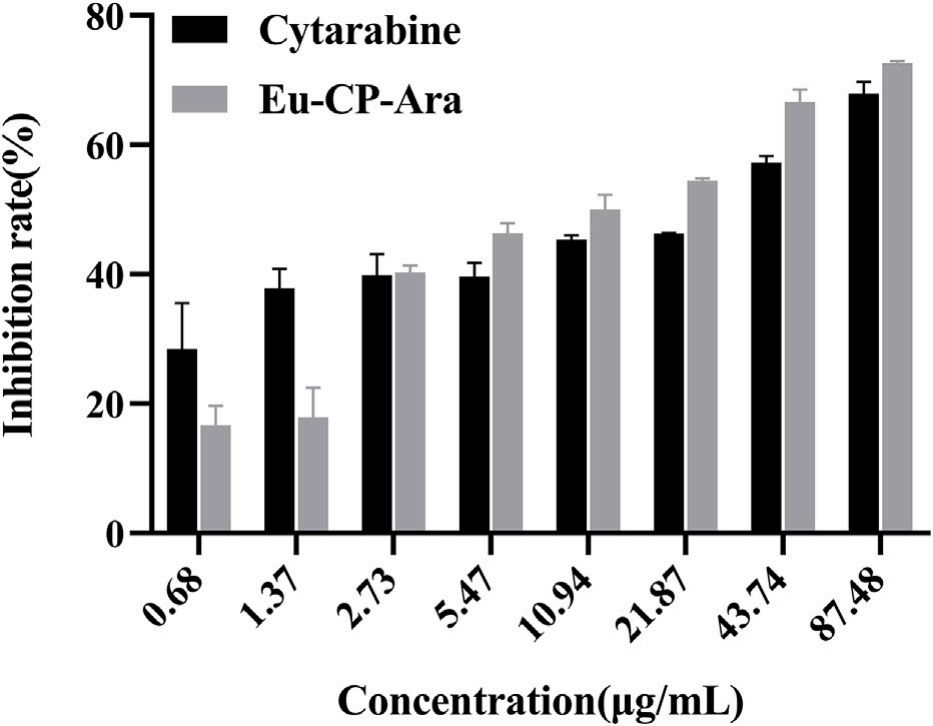
Inhibitory rates of cytarabine and Eu-CP-Ara at different concentrations on A549 cells (0.68, 1.37, 2.73, 5.47, 10.94, 21.87, 43.74, and 87.48 μg/ml).

**FIGURE 9:**
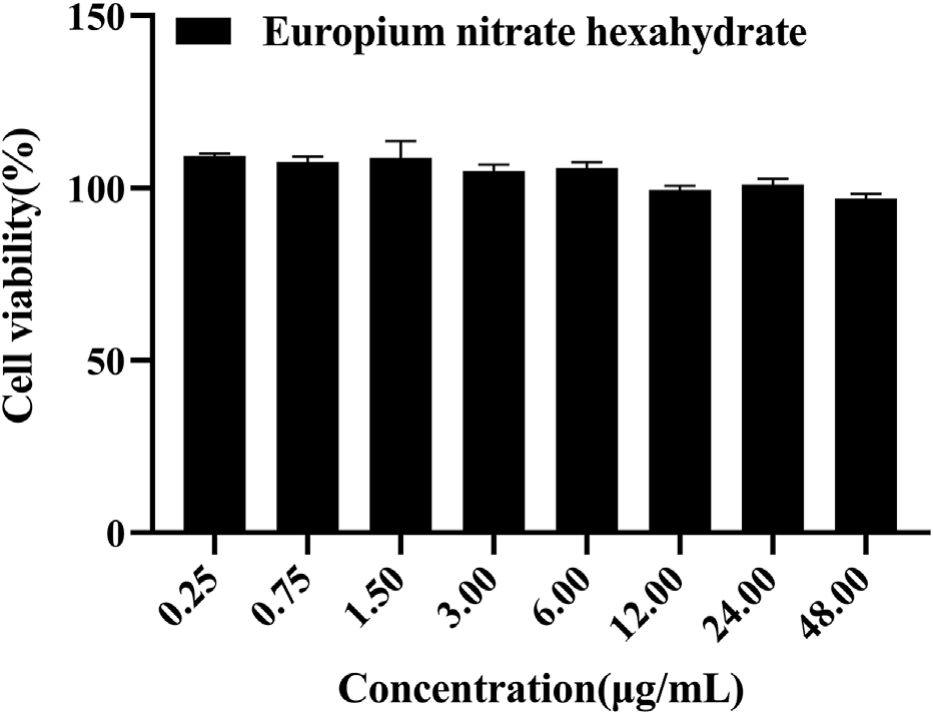
Cell viability of europium nitrate hexahydrate at different concentrations on A549 cells (0.25, 0.75, 1.5, 3, 6, 12, 24, and 48 μg/ml).

**FIGURE 10:**
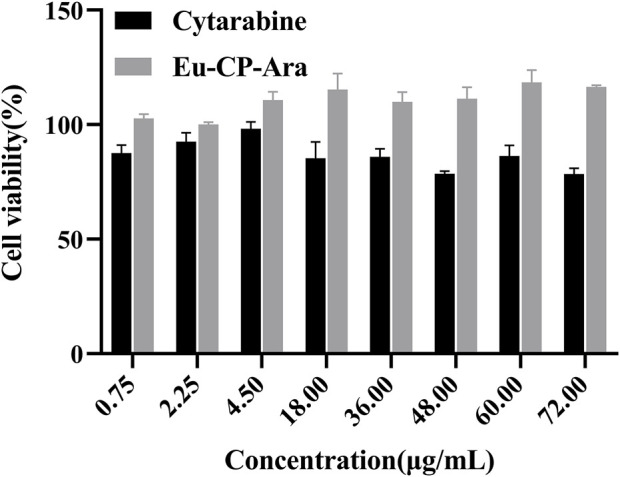
Cell viability of cytarabine and Eu-CP-Ara at different concentrations on L02 cells (0.75, 2.25, 4.5, 18, 36, 48, 60, and 72 μg/ml).

**FIGURE 11:**
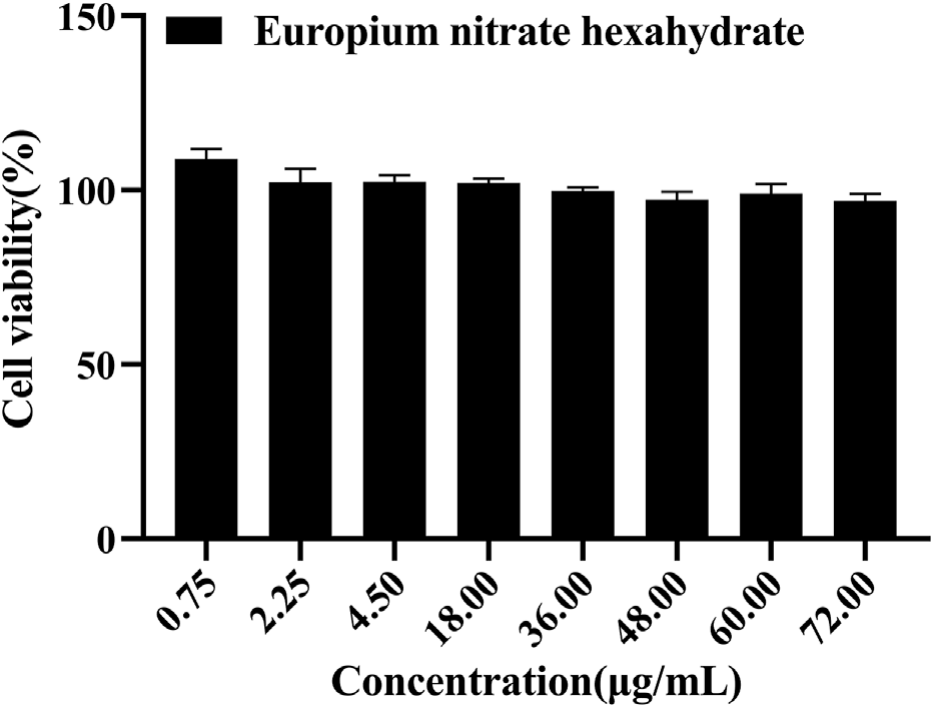
Cell viability of europium nitrate hexahydrate at different concentrations on L02 cells (0.75, 2.25, 4.5, 18, 36, 48, 60, and 72 μg/ml).

Cytotoxicity experiments the cytotoxicity of cytarabine with Eu-CP-Ara against A549 was investigated (Figure 8). In addition, the cell viability of A549 and L02 were tested, respectively, in the presence of cytarabine, Eu-CP-Ara and europium nitrate hexahydrate (Figures 9–11). As shown in Figure 8, it is obvious to see that there is a large difference in the inhibition rate of cytarabine *versus* Eu-CP-Ara on A549 cell viability. We can easily draw a conclusion that both Cytarabine and Eu-CP-Ara have strong inhibition on this cell, and the cell inhibition rate was positively correlated with the drug concentration (Zeng et al., 2020). Proliferation of A549 was almost unaffected in the presence of equivalent europium nitrate hexahydrate alone. This suggests that europium ions may have a synergistic effect with cytarabine to produce a stronger toxic effect on tumor cells. And, Eu-CP Ara had lower cytotoxicity against L02 cells.

## Conclusion

In summary, Eu-CP-Ara micro/nanospheres with luminescence properties, good bioactivity, good biocompatibility and good dispersibility in water have been successfully prepared for our study. The results have demonstrated that the luminescence properties wavelength of Eu-CP-Ara is emission centering at about 619 nm. Meanwhile, Eu-CP-Ara has stronge inhibitory effect on the activity of A549 cells and lower cytotoxicity to normal liver cells (L02). Based on these results, Eu-CP-Ara can be used as a potential anticancer drug. The combination of this luminescence properties, Eu-CP-Ara may be an effective strategy for the tracking cytarabine against tumours and might impart better accurate treatment effect and therapeutic efficiency. The use of micro/nanoparticle-based formulations and cell pharmacodynamic experiment might provide a novel topical therapeutic approach. As for their antitumor activity and luminescence properties, wide bioapplications may be found.

## Data Availability

The original contributions presented in the study are included in the article/Supplementary Material, further inquiries can be directed to the corresponding author.
